# Supplementary material for: Understanding the effect of human capital and decent work for migrants’ integration using PLS-SEM
Source: PLoS One. 2025 Apr 29;20(4):e0322196. doi: 10.1371/journal.pone.0322196 (PMC12040112; doi:10.1371/journal.pone.0322196)
Supplement: Attachment — Submitted filename: PLOS_ONE_12Mar_Letter.pdf [file pone.0322196.s002.pdf]

RESPONSE LETTER

Manuscript ID: PONE-D-24-51928

Title: Understanding the effect of human capital and decent work for migrants' integration using PLS-SEM

Dear Professor Dr. Voxi Heinrich Amavilah, Ph.D

Editor Dr. Voxi Heinrich Amavilah, Ph.D

Thank you very much for considering our research paper for possible publication in the esteemed Plos One. We are grateful for your oversight of this process and the reviewers' suggestions to improve our revised submission. We have made all suggested revisions and we appreciate the reviewers' suggestions and calls for more clarification regarding our submission. After careful consideration, we now resubmit this paper for your review.

This response letter addresses all the revisions as indicated in the reviewers' comments prefixed as “COMMENT:”, while our responses are prefixed with “AUTHORS' RESPONSE:”.

We hope that this submission of our paper meets the quality standard and expectations of the Plos One editors, reviewers, and readers.

Sincerely,

Valentina Gomes Haensel Schmitt

Corresponding author

“COMMENT”
“PONE-D-24-51928
Understanding the effect of human capital and decent work for migrants’ integration using PLS-SEM
PLOS ONE

Dear Dr. Schmitt,

Thank you for submitting your manuscript to PLOS ONE. After careful consideration, we feel that it has merit but does not fully meet PLOS ONE’s publication criteria as it currently stands. Therefore, we invite you to submit a revised version of the manuscript that addresses the points raised during the review process.

Please submit your revised manuscript by Mar 15 2025 11:59PM. If you will need more time than this to complete your revisions, please reply to this message or contact the journal office at plosone@plos.org. When you're ready to submit your revision, log on to https://www.editorialmanager.com/pone/ and select the 'Submissions Needing Revision' folder to locate your manuscript file.

Please include the following items when submitting your revised manuscript:

- A rebuttal letter that responds to each point raised by the academic editor and reviewer(s). You should upload this letter as a separate file labeled 'Response to Reviewers'.
- A marked-up copy of your manuscript that highlights changes made to the original version. You should upload this as a separate file labeled 'Revised Manuscript with Track Changes'.
- An unmarked version of your revised paper without tracked changes. You should upload this as a separate file labeled 'Manuscript'.

If you would like to make changes to your financial disclosure, please include your updated statement in your cover letter. Guidelines for resubmitting your figure files are available below the reviewer comments at the end of this letter.

If applicable, we recommend that you deposit your laboratory protocols in protocols.io to enhance the reproducibility of your results. Protocols.io assigns your protocol its own identifier (DOI) so that it can be cited independently in the future. For instructions see: https://journals.plos.org/plosone/s/submission-guidelines#loc-laboratory-protocols. Additionally, PLOS ONE offers an option for publishing peer-reviewed Lab Protocol articles, which describe protocols hosted on protocols.io. Read more information on sharing protocols at https://plos.org/protocols?utm_medium=editorial-email&utm_source=authorletters&utm_campaign=protocols.

We look forward to receiving your revised manuscript.

Kind regards,

Voxi Heinrich Amavilah, Ph.D.
Academic Editor
PLOS ONE

“AUTHORS’ RESPONSE:”
Dear Editor,
Once again, we would like to thank you for allowing the revision and resubmission of our study. As you will note, we had proceeded with all the required revisions from Reviewer 1 and 2. As requested, we are uploading at the system the required documents: 1. The rebuttal letter; 2. A marked-up copy of our manuscript; 3. An unmarked version of our manuscript.
Besides, at the letter, we are providing at this document the explanation each presented comment. We hope it reaches both the reviewer´s and editor´s expectation for this works publication.
We look forward for receiving the feedback.
Best regards,

Mirza Cequea, Jessika Vasquez and Valentina Schmitt.

“COMMENT”

Journal Requirements:

When submitting your revision, we need you to address these additional requirements.

1. Please ensure that your manuscript meets PLOS ONE's style requirements, including those for file naming. The PLOS ONE style templates can be found at

https://journals.plos.org/plosone/s/file?id=wjVg/PLOSOne_formatting_sample_main_body.pdf and https://journals.plos.org/plosone/s/file?id=ba62/PLOSOne_formatting_sample_title_authors_affiliations.pdf

“AUTHORS’ RESPONSE:”

The manuscript format was modified to align with PLOS ONE's style guidelines.

“COMMENT”

2. Please note that your Data Availability Statement is currently missing the repository name. If your manuscript is accepted for publication, you will be asked to provide these details on a very short timeline. We therefore suggest that you provide this information now, though we will not hold up the peer review process if you are unable.

“AUTHORS’ RESPONSE:”

The data used in the preparation of this article are stored and available as Open Access, without restriction for it use, at the following address:
“Data for the project: Capital Humano y Trabajo Decente: Migrantes venezolanos en el Perú como sujetos productivos el mercado laboral el Caso Lima Metropolitana”. https://doi.org/10.7910/DVN/UHGMYG.

“COMMENT”

3. Please include a separate caption for each figure in your manuscript.

“AUTHORS’ RESPONSE:”

We´ve proceeded to provide a separate caption for each figure in our manuscript.

“COMMENT”

4. Please review your reference list to ensure that it is complete and correct. If you have cited papers that have been retracted, please include the rationale for doing so in the manuscript text, or remove these references and replace them with relevant current references. Any changes to the reference list should be mentioned in the rebuttal letter that accompanies your revised manuscript. If you need to cite a retracted article, indicate the article’s retracted status in the References list and also include a citation and full reference for the retraction notice.

“AUTHORS’ RESPONSE:”

We´ve double check to make sure it is complete and correct.

“COMMENT”

Additional Editor Comments:

Reviewer 2 has given you comments that would greatly improve your MS; please respond to them thoroughly. Like Reviewer 1, I would like to see some assessment of the wider implications of this Peruvian case study.

“AUTHORS’ RESPONSE:”

Dear Editor, once again, we would like to thank you for taking the time to manage the review thoroughly our material. We have considered all recommendations and added them to this recent version. We hope it now meets all your expectation.

Reviewers' comments:

Reviewer's Responses to Questions

Comments to the Author

“COMMENT”

1. Is the manuscript technically sound, and do the data support the conclusions?

The manuscript must describe a technically sound piece of scientific research with data that supports the conclusions. Experiments must have been conducted rigorously, with appropriate controls, replication, and sample sizes. The conclusions must be drawn appropriately based on the data presented.

Reviewer #1: Yes

Reviewer #2: Yes

“AUTHORS’ RESPONSE:”
We would like to thank both reviewers for recognizing it.

“COMMENT”

2. Has the statistical analysis been performed appropriately and rigorously?

Reviewer #1: Yes

Reviewer #2: Yes

“AUTHORS’ RESPONSE:”
We would like to thank both reviewers for recognizing it.

“COMMENT”

3. Have the authors made all data underlying the findings in their manuscript fully available?

The PLOS Data policy requires authors to make all data underlying the findings described in their manuscript fully available without restriction, with rare exception (please refer to the Data Availability Statement in the manuscript PDF file). The data should be provided as part of the manuscript or its supporting information, or deposited to a public repository. For example, in addition to summary statistics, the data points behind means, medians and variance measures should be available. If there are restrictions on publicly sharing data— e.g. participant privacy or use of data from a third party—those must be specified.

Reviewer #1: Yes

Reviewer #2: Yes

“AUTHORS’ RESPONSE:”
We would like to thank both reviewers for recognizing it.

“COMMENT”

4. Is the manuscript presented in an intelligible fashion and written in standard English?
PLOS ONE does not copyedit accepted manuscripts, so the language in submitted articles must be clear, correct, and unambiguous. Any typographical or grammatical errors should be corrected at revision, so please note any specific errors here.

Reviewer #1: Yes

Reviewer #2: Yes

“AUTHORS’ RESPONSE:”
We would like to thank both reviewers for recognizing it.

5. Review Comments to the Author

Please use the space provided to explain your answers to the questions above. You may also include additional comments for the author, including concerns about dual publication, research ethics, or publication ethics. (Please upload your review as an attachment if it exceeds 20,000 characters)

Reviewer #1: The article is prepared at a fairly high level. The research topic is important and relevant.

“COMMENT”
It is worth adding an assessment of the representativeness of the sample of respondents.
“AUTHORS’ RESPONSE:”
We´ve added the following information to the file:
“The data were taken from Cequea et al. (2024), so they were secondary data. The authors noted, with respect to the sample size “it was considered that 889,809 Venezuelans resided in Lima …, with a margin of error of 3 % and a confidence level of 95 %, having an estimated sample size of 1067 subjects. However, this study received a total of 1193 valid responses to the survey and 98 invalid ones.”(p. 5). However, the number of valid surveys exceeded the sample size, so it is considered that this sample size overcomes the drawbacks of non-probability sampling used for data collection in this study.”

“COMMENT”
It is worth adding the possibility of using the results in a business environment.
“AUTHORS’ RESPONSE:”

Thank you for your recommendation. We´ve added it to the new version.

“COMMENT”

Reviewer #2: Review of Manuscript PONE-D-24-51928

Title: Understanding the Effect of Human Capital and Decent Work for Migrants' Integration Using PLS-SEM

General Comments:

This manuscript addresses a critical and timely issue: the integration of Venezuelan migrants in Peru, focusing on the roles of human capital and decent work. The study utilizes Partial Least Squares Structural Equation Modeling (PLS-SEM) to explore the relationships among these variables, providing insights relevant for policymakers and stakeholders in migration and labor markets. The manuscript is well-structured, and its findings are significant for understanding migrant integration in contexts characterized by labor informality. However, there are several areas where the manuscript could be improved to enhance its clarity, rigor, and impact.

“AUTHORS’ RESPONSE:”

Dear Reviewer, we would like to extend our sincere gratitude for dedicating your time and expertise to evaluate our manuscript. Your insightful comments and constructive feedback have been instrumental in enhancing the quality and rigor of our work.

We have carefully considered each of the points raised, and in response, we have made the necessary revisions to address the concerns raised. We are confident that these revisions align with your expectations and have effectively clarified pertinent aspects of the manuscript.

We greatly value your contributions in ensuring the scholarly rigor and integrity of our research. We believe that the revisions made have strengthened the manuscript and we look forward to your further guidance as we move towards the publication stage.

Thank you once again for your invaluable input and for the opportunity to contribute to the body of knowledge in our field.

Specific Comments:

“COMMENT”

1. Research Context and Contribution:

   Strengths: The study's focus on Venezuelan migrants in Peru addresses a gap in the migration literature, particularly in the context of Latin America's high labor informality. The emphasis on decent work as a mediating factor is innovative and aligns well with the United Nations’ Sustainable Development Goals.

   Suggestions for Improvement: While the manuscript discusses the relevance of the study, it could provide a more detailed comparison with similar studies in other regions to highlight its unique contribution further. For example, are there lessons from other countries facing similar migration challenges that could enrich the discussion?

“AUTHORS’ RESPONSE:”

Thank you for the recommendation. We´ve added the following statement to the text:

“The literature review showed that there are numerous studies that relate human capital or the high skills of migrants with obtaining fair working conditions or decent work and this in turn as a facilitator of integration into the host society (); however, no studies were found that propose a direct relationship between human capital and integration considering decent work as a mediating variable, nor studies that employ structural equations or causal relationships between these constructs.

Peru's neighboring countries face similar migration challenges; but, although Venezuelans migrate fleeing violence, seeking work and improvements in their quality of life, the region is known for its high rates of informal employment, being this a structural problem, the incidence of this labor informality among Venezuelans abroad is particularly high. In Peru, 97% of work is in the informal sector, while in Panama, Ecuador, Colombia and Trinidad and Tobago, the Venezuelan labor force

faces between 73% and 89% (R4V, 2025). Informality usually implies long working hours, lower wages, absence of social protection and related benefits, and often unsafe and precarious working conditions. In this context, various measures have been implemented in neighboring countries where Venezuelan migration has overwhelmed the capacity of local governance. To address this phenomenon, the Interagency Coordination Platform for Refugees and Migrants (R4V) was created to coordinate efforts under the Refugee and Migrant Response Plan for Venezuela (RMRP) in 17 countries in Latin America and the Caribbean. Countries such as Colombia, Ecuador and Chile, the main destinations of Venezuelan migrants and Peru's neighbors, have implemented various migration control strategies. These strategies include migration control policies, focused on regulating border flows; regularization, aimed at granting legal status to migrants; and security, focused on measures related to national and border security (R4V, 2025)."

2. Methodology:

Strengths: The use of PLS-SEM is appropriate for the study's objectives and the complexity of the model. The detailed description of the data analysis process, including measures to address common method bias (CMB) and validate the model, is commendable.

"COMMENT"

Suggestions for Improvement:

Clarify the rationale for selecting the specific constructs and indicators used for human capital, decent work, and integration. Were these based on prior validated scales, or were they developed specifically for this study?

"AUTHORS' RESPONSE:"

Thank you for the recommendation. We´ve added the following statement to the text: "The constructs and dimensions used to assess human capital, decent work and integration were based on previously validated scales and previously established theoretical frameworks (Cequea et al., 2024b)."

“COMMENT”

Provide more details on the demographic characteristics of the sample, particularly regarding gender, age, and education levels. While Figure 2 provides an overview, a more detailed breakdown would enhance the reader's understanding of the sample's representativeness.

“AUTHORS’ RESPONSE:”

This section was rewritten to provide more detail on the characteristics of the sample, as suggested. It may be noted at the text, as follows:

“Figure 2 provides a detailed overview of the sample's demographic characteristics. Women represent the majority of the sample (62%), while men comprise 38%. Regarding age distribution, the largest group falls within the 31–40 age range (37% for both genders), followed by participants aged 21–30 **(**28% of women and 34% of men**).** A smaller proportion of participants are above 50, with only 6% of men and 10% of women in the 51–60 age group, and even fewer aged 61 or older.

Marital status indicates that single individuals dominate the sample, accounting for 70% of women and 66% of men**.** Married participants represent 18% of women and 22% of men, while divorced and widowed individuals make up a small minority.

In terms of education, the sample reflects high human capital. Among women, 41% hold university degrees, compared to 34% of men. Postgraduate qualifications are less common, with 6% of women and 3% of men attaining this level. A notable proportion of men have secondary (40%) or technical education (22%), compared to 32% and 21% of women, respectively.

Employment patterns reveal a significant prevalence of independent work, particularly among women (46%) compared to men (36%). Meanwhile, 37% of women and 43% of men are employed without formal contracts, underscoring the informal nature of employment within the sample. A smaller subset of participants is employed under contractual agreements, with slightly more men (22%) than women (18%).

Residency duration in Peru varies across the sample. Most participants have lived in the country for three to five years, with 32% of women and 29% of men reporting 3–4 years of residence, and 28% of women and 39% of men indicating 4–5 years. These patterns suggest a mix of recently arrived individuals and those who are more established, which impacts labor market integration and stability.

Additional data highlight that 65% of respondents have 1–10 years of professional experience, indicating a relatively experienced workforce. Work intensity is notable, with 59% of participants working six days per week and 38% reporting shifts of 10–12 hours daily. This reflects the demanding nature of their work environments and economic necessity.

Lastly, 51% of respondents report practicing their profession in Peru, demonstrating a level of professional integration. However, this also implies that nearly half face barriers in exercising their qualifications, likely due to issues related to credential recognition or occupational mismatch."

"COMMENT"

Discuss the potential limitations of using secondary data, particularly in terms of its applicability to the current research questions.

"AUTHORS' RESPONSE:"

This discussion was incorporated into the item "**6.2. Limitations and future research directions**", as follows:

"A limitation of secondary research is that the data were collected for another purpose and may not fully meet the needs of the current research. The objectives and methodology used to collect the primary data may not be appropriate for the problem at hand, so the quality of the data source must be carefully reviewed (Schiffman & Kanuk, 2005; Heinemann, 2007). For example, in the primary data collection (Cequea et al., 2024a), the data collection technique used did not guarantee parity between men and women, and the sampling was not random, so it is not possible to ensure representativeness. This could make it difficult to reach more robust conclusions if the objective of our study were aimed at making comparisons between groups of men and women; however, this was not the objective of the present study. On the other hand, to avoid assuming different meanings of the variables used for this secondary study, we reviewed the conceptualization and operationalization of these variables in the primary study (Cequea et al., 2024b) and adopted them for the secondary study."

3. Results:

“COMMENT”

Strengths: The findings are clearly presented and supported by robust statistical analyses. The use of bootstrapping to validate the mediation analysis adds credibility to the results.

“AUTHORS’ RESPONSE:”

We would like to thank both reviewers for recognizing it.

“COMMENT”

Suggestions for Improvement:

Expand on the implications of the low mediation effect of decent work. While the study acknowledges Peru's high labor informality, it would be helpful to explore additional factors that might explain this limited effect.

“AUTHORS’ RESPONSE:”

Thank you for the recommendation. We´ve added the following statement to the text: “According to the International Labor Organization (ILO, 1999), decent work implies access to productive opportunities in conditions of freedom, equity, security and human dignity, essential aspects for the social and economic integration of migrants in host societies, since it not only guarantees stable income and decent working conditions, but also promotes their social recognition, civic participation and general well-being. Likewise, access to employment with fair conditions allows migrants to develop economic autonomy, facilitating their stability and access to housing, education and health services. Additionally, it encourages the use of skills and knowledge acquired in the country of origin, avoiding overqualification and waste of talent, which reinforces their self-esteem and sense of belonging (Portes & Rumbaut, 2014). Finally, the workplace is a space where migrants can establish contact networks, learn cultural norms and develop interpersonal relationships that favor their social integration (Ager & Strang, 2008). In this sense, inclusive labor policies strengthen social cohesion and economic stability, benefiting both migrants and society as a whole, since when host countries guarantee fair labor conditions, they not only protect migrants' rights, but also take advantage of their human capital and promote more cohesive and equitable societies (Dustmann & Glitz, 2011).

Another aspect that could be affecting the low mediation of decent work are the barriers faced by migrants in Peru to regularize their qualifications and validate their knowledge and experience, finding themselves limited to underemployment and not being able to access well-paid jobs commensurate with their skills, which reduces their quality of life."

"COMMENT"

Include a discussion on the potential differences in integration outcomes for various subgroups within the sample (e.g., by gender, age, or length of residency).

"AUTHORS' RESPONSE:"

First of all, we would to thank you for the valuable recommendation. At this point, we would like to inform you that we were unable to respond to your suggestion due to not having access to the PLS software.

4. Discussion and Policy Implications:

Strengths: The discussion effectively links the findings to broader policy implications, emphasizing the need for labor formalization and recognition of migrant skills.

Suggestions for Improvement:

"COMMENT"

Provide specific examples of policies or programs from other countries that have successfully addressed similar challenges. This would strengthen the practical relevance of the recommendations.

"AUTHORS' RESPONSE:"

We´ve incorporated further explanation and examples from Colombia, Chile and Ecuador, as follows:

"Challenges for implementing labor formalization policies in the Peruvian context:

Despite the need for policies that promote labor formalization to facilitate migrant integration, their implementation in Peru faces significant structural challenges. High

labor informality reflects not only a lack of access to formal jobs but also issues such as bureaucracy and the lack of economic incentives for employers and workers. For instance, the high costs associated with formally registering employees, along with the administrative burden of complying with complex labor regulations, discourages many companies, especially small and medium-sized ones, from formalizing their workforce. These barriers disproportionately affect migrants, who often lack the social networks or resources to navigate these processes effectively. Moreover, the prevalence of informal employment reduces opportunities for skilled migrants to access jobs that align with their qualifications, limiting their potential contribution to the formal economy. Additionally, the lack of an efficient system for recognizing foreign degrees and professional experience remains a significant barrier to the integration of skilled migrants. Without a streamlined accreditation process, many highly qualified migrants are unable to practice their professions, fostering brain waste and limiting their economic contribution. This challenge is particularly acute in sectors such as health and education, where accreditation is essential for employment.

Strategies to Overcome These Challenges:

To address these challenges, several strategies could be implemented. First, simplifying administrative procedures and reducing the costs associated with formal employee registration could encourage more companies to formalize their workforce. Examples from countries such as Colombia and Chile, which have implemented tax incentives and simplified digital platforms for formalization, could serve as models for Peru (Weller, 2009; Arredondo & Concha, 2021). Or strategies for rapid regularization of their stay in the context of arrival, as in the case of Ecuador, so that they can access formal employment, the public health system, rental housing and banking, among others (Herrera & Cabezas, 2019).This might include creating tax incentive programs for employers who formally hire skilled migrants, as well as subsidies to cover part of the formalization costs in strategic sectors.

Second, creating a flexible and accessible accreditation system to recognize foreign degrees would be essential. This could be achieved through cooperation agreements with foreign universities and educational institutions, as well as through the development of practical assessments that allow migrants to directly demonstrate their competencies.

Finally, establishing centralized digital platforms where migrants can access information on formalization opportunities and legal resources would simplify the

process, especially in areas where access to technological infrastructure is limited. Collaboration between the government, NGOs, and the private sector could facilitate the dissemination and access to these resources, thus promoting a more effective and productive integration. Such platforms could also serve as hubs for professional networking and mentorship opportunities, which are critical for skilled migrants navigating new labor markets."

"COMMENT":

Discuss potential barriers to implementing the proposed policies in Peru and suggest strategies to overcome these challenges.

"AUTHORS' RESPONSE:"

This point may be noted at the following statements:

"The results confirm hypothesis H2, showing that migrants' human capital is a determining factor in obtaining decent jobs. Migrants with higher education and experience levels are more likely to access formal and stable jobs (Cui et al., 2022). However, the results also reveal that many migrants in Peru, despite being highly qualified, are forced to work in informal jobs, limiting their development and reflecting a brain waste phenomenon. This finding underscores the need for policy interventions that promote labor formalization and remove barriers to decent employment for skilled migrants, thus allowing them to realize their potential in the local economy…

However, barriers to integration and the prevalence of precarious working conditions can lead to brain waste, limiting both the economic contributions and social integration of skilled migrants (Bhardwaj and Sharma, 2023). Policies aimed at bridging these gaps are essential to realize the dual benefits of human capital development and migrant integration…

Another aspect that could be affecting the low mediation of decent work are the barriers faced by migrants in Peru to regularize their qualifications and validate their knowledge and experience, finding themselves limited to underemployment and not being able to access well-paid jobs commensurate with their skills, which reduces their quality of life…

These barriers disproportionately affect migrants, who often lack the social networks or resources to navigate these processes effectively. Moreover, the prevalence of informal employment reduces opportunities for skilled migrants to

access jobs that align with their qualifications, limiting their potential contribution to the formal economy…

Additionally, the lack of an efficient system for recognizing foreign degrees and professional experience remains a significant barrier to the integration of skilled migrants. Without a streamlined accreditation process, many highly qualified migrants are unable to practice their professions, fostering brain waste and limiting their economic contribution. This challenge is particularly acute in sectors such as health and education, where accreditation is essential for employment.

5.6 Strategies to Overcome These Challenges…"

5. Ethical Considerations:

Strengths: The manuscript appropriately addresses ethical issues, including the use of secondary data from a publicly available database and approval by an ethics committee.

"COMMENT"

Suggestions for Improvement: Reiterate the steps taken to ensure the anonymity and confidentiality of participants, especially given the sensitive nature of migration data.

"AUTHORS' RESPONSE:"

At this point, we would like to mention that the database we have used is public and all data was anonymized to ensure the confidentiality of the participants.

This study was based on secondary data and therefore does not raise ethical issues regarding the participation of human subjects. The data used originated from the study by Cequea et al. (2024a), which was reviewed and approved by the ethics committee of the University of Lima, Peru (Cequea et al., 2024b). According to Cequea et al. (2024b), the data set was anonymized during collection, with all personally identifiable information removed, ensuring confidentiality and protection of the participants' data. Before completing the questionnaire, participants gave written consent, as required by ethical protocols. The study received ethical approval from the ethics committee of the University of Lima -Dictamen N. 027-CEI-IDIC-IDIC-ULIMA-2022- (Cequea et al., 2024b). By using secondary data from a publicly available and ethically reviewed source, this study adheres to international ethical standards for

research, including the Declaration of Helsinki. These measures ensure the safeguarding of sensitive migration data and address potential ethical issues related to participant confidentiality.

6. Writing and Presentation:

Strengths: The manuscript is well-written and follows a logical structure, making it easy to follow the arguments and findings.

Suggestions for Improvement:

“COMMENT”
Simplify technical terms and provide additional explanations for readers unfamiliar with PLS-SEM.
“AUTHORS’ RESPONSE:”
Following this recommendation, we have modified the explanation of data analysis and method bias in order to make it more understandable for those unfamiliar with PLS-SEM, as follows:

“Data analysis. The data analysis process involved several steps to ensure data quality and robust results. After cleaning the data by removing outliers and imputing missing values, the study applied Partial Least Squares Structural Equation Modeling (PLS-SEM). This technique is particularly suited for predictive research involving complex models with latent variables. PLS-SEM was selected for its flexibility in handling non-normal data distributions—a characteristic often observed in datasets from migrant populations—and its ability to analyze multiple dependent variables simultaneously. These features align with the study's objective of exploring the interconnected effects of human capital, decent work, and integration (Ringle et al., 2012; Hair et al., 2022a).

To enhance the predictive validity of the findings, the study employed PLSpredict, a procedure used to evaluate the model's predictive relevance by generating predictions for unseen data, focusing on Q^2 and Standardized Root Mean Square Residual (SRMR) as key indicators. Q^2 measures the model’s predictive relevance, while SRMR evaluates its fit by examining the residuals between observed and

predicted correlations. In this study, SRMR values remained within acceptable thresholds, confirming the model’s ability to accurately represent the data structure. This analysis validated both the relationships between variables and the model’s predictive accuracy for new data (Hair et al., 2022b; Sarstedt et al., 2019).

The study adhered to established PLS-SEM evaluation guidelines (Hair et al., 2022a; 2022b; Ringle et al., 2020). Both the measurement model – which evaluates construct reliability and validity – and the structural model – which examines relationships between variables – were rigorously tested. This comprehensive approach enhances the policy relevance and practical applicability of the findings for migrant integration.

Addressing Common Method Bias (CMB). To address potential Common Method Bias (CMB) and ensure research validity, the study implemented preventive measures during its design phase, following the recommendations of Schwarz et al. (2017). Additionally, statistical tests such as the lateral collinearity test confirmed that CMB did not significantly influence the results (MacKenzie and Podsakoff, 2012). The analysis yielded a variance inflation factor (VIF) of 1.268, well below the threshold of 3.3 (Kock & Lynn, 2012), further validating the robustness of the model.”

“COMMENT”

Ensure consistency in the use of terms like "human capital," "decent work," and "integration." A glossary or definition section could be helpful.

“AUTHORS’ RESPONSE:”

We have provided and highlighted the definition, as follows:

“Human capital is understood as the set of productive skills and knowledge of a population, with educational level being a key indicator of this capital (Cui et al., 2022; Rajendran et al., 2017; Tornos et al., 2004).”

Decent work is defined as productive employment that provides fair remuneration, security, social protection, and equal treatment (Alzola, 2018; ILO, 2019; Mieres, 2024; Mishchuk et al., 2021; Sazali and Tumin, 2020). This concept is a fundamental goal of the United Nations 2030 Agenda (Sazali and Tumin, 2020) and is particularly relevant for migrants, displaced persons, and refugees, who need their labor rights to be respected in host countries (Piper et al., 2017).”

“Integration involves not only labor market insertion but also access to housing, education, healthcare services, and the exercise of civic rights (Piper et al., 2017).

According to Khan and Sandhu (2016), promoting decent work practices and understanding migrant cultures can improve migration policies and facilitate their integration.

Social integration represents a sense of belonging and cohesion, achieved through cultural adaptation and the creation of support networks (Teixeira et al., 2021; Tharmaseelan et al., 2010). Economic integration, on the other hand, is facilitated by stable employment that allows for the coverage of basic needs and provides financial stability (Blouin, 2019). Fair remuneration, as a central element of decent work, contributes to improving quality of life and supports an effective integration process (Lukas, 2021)."

Additional Comments on Dual Publication, Research Ethics, or Publication Ethics:

"COMMENT"

Dual Publication: Ensure that no portion of this manuscript has been previously published or is under consideration elsewhere. While secondary data usage is appropriate, confirm that the authors have not presented identical analyses in other publications.

"AUTHORS' RESPONSE:"

Thank you for your concern. Regarding this point, we may assure that no portion of this manuscript has been previously published or is under consideration elsewhere, and we do not present identical analyses in other publications.

"COMMENT"

Research Ethics: The study relies on secondary data from a publicly available source. While the manuscript notes that consent was obtained, the authors should clarify whether additional permissions were required to reuse this dataset for the current study.

"AUTHORS' RESPONSE:"

The data used in the preparation of this article are stored and available as Open Access, without restriction for it use, at the following address:

“Data for the project: Capital Humano y Trabajo Decente: Migrantes venezolanos en el Perú como sujetos productivos el mercado laboral el Caso Lima Metropolitana”. https://doi.org/10.7910/DVN/UHGMYG

“COMMENT”

Publication Ethics: The manuscript appears to meet the ethical standards required for publication. However, the authors should confirm that all co-authors have approved the final manuscript and disclose any potential conflicts of interest.

“AUTHORS’ RESPONSE:”

All co-authors have approved the final manuscript and disclosed no potential conflicts of interest.

“COMMENT”

Recommendation:

Based on the above evaluation, I recommend:

Minor Revisions to address the suggestions outlined above. The manuscript has significant potential, and these revisions will enhance its clarity, rigor, and relevance.

Summary of Suggestions:

- Provide a more detailed comparison with similar studies in other regions. Clarify the rationale for construct selection and address potential limitations of secondary data.
- Expand on subgroup analysis and implications of the mediation effect.
- Include examples of successful policies from other countries and discuss barriers to implementation in Peru.
- Simplify technical terms and ensure consistency in terminology.
- Confirm compliance with ethical standards and provide additional details on data reuse.

The findings of this study have the potential to contribute meaningfully to the field of

migration and labor economics, particularly in the context of Latin America. Addressing the suggestions outlined above will strengthen the manuscript and enhance its impact.

“AUTHORS’ RESPONSE:”

Once again, we would like to thank you for your thoroughly effort on revising it. Your contributions allow us to present a better and improved version of our study. Best regards, the authors.

“COMMENT”

6. PLOS authors have the option to publish the peer review history of their article (what does this mean?). If published, this will include your full peer review and any attached files.

If you choose “no”, your identity will remain anonymous but your review may still be made public.

Do you want your identity to be public for this peer review? For information about this choice, including consent withdrawal, please see our Privacy Policy.

Reviewer #1: No

Reviewer #2: **Yes:** DEJENDRAN RAJENRAN

“AUTHORS’ RESPONSE:”

Once again, we would like to thank both reviewers for your thoroughly effort on revising it.

“COMMENT”

[NOTE: If reviewer comments were submitted as an attachment file, they will be attached to this email and accessible via the submission site. Please log into your account, locate the manuscript record, and check for the action link "View Attachments". If this link does not appear, there are no attachment files.]

While revising your submission, please upload your figure files to the Preflight Analysis and Conversion Engine (PACE) digital diagnostic tool, https://pacev2.apexcovantage.com/. PACE helps ensure that figures meet PLOS requirements. To use PACE, you must first register as a user. Registration is free. Then, login and navigate to the UPLOAD tab, where you will find detailed instructions on how to use the tool. If you encounter any issues or have any questions when using PACE, please email PLOS at figures@plos.org. Please note that Supporting Information files do not need this step.

"AUTHORS' RESPONSE:"

Thank you for the recommendation and support. We had proceeded to revise the figure files to the Preflight Analysis and Conversion Engine (PACE) digital diagnostic tool to ensure it meets PLOS requirements.
